# Establishment of an in vitro model of monocyte-like THP-1 cells for trained immunity induced by bacillus Calmette-Guérin

**DOI:** 10.1186/s12866-024-03191-x

**Published:** 2024-04-20

**Authors:** Jin-Chuan Xu, Kang Wu, Rui-qing Ma, Jian-hui Li, Jie Tao, Zhidong Hu, Xiao-Yong Fan

**Affiliations:** 1 Shanghai Public Health Clinical Center & Shanghai Institute of Infectious Diseases and Biosecurity, Fudan University, Shanghai, China; 2 Shanghai R & S Biotech. Co., Ltd, Shanghai, China; 3 Zhejiang Free Trade Area R & S Biomedical Technology Co., Ltd, Zhoushan, Zhejiang China

**Keywords:** Monocyte, THP-1, Bacillus Calmette-Guérin (BCG), Trained immunity

## Abstract

**Background:**

Mycobacteria bloodstream infections are common in immunocompromised people and usually have disastrous consequences. As the primary phagocytes in the bloodstream, monocytes and neutrophils play critical roles in the fight against bloodstream mycobacteria infections. In contrast to macrophages, the responses of monocytes infected with the mycobacteria have been less investigated.

**Results:**

In this study, we first established a protocol for infection of non-adherent monocyte-like THP-1 cells (i.e. without the differentiation induced by phorbol 12-myristate 13-acetate (PMA) by bacillus Calmette-Guérin (BCG). Via the protocol, we were then capable of exploring the global transcriptomic profiles of non-adherent THP-1 cells infected with BCG, and found that NF-κB, MAPK and PI3K-Akt signaling pathways were enhanced, as well as some inflammatory chemokine/cytokine genes (*e.g. CCL4*, *CXCL10*, *TNF* and *IL-1β*) were up-regulated. Surprisingly, the Akt-HIF-mTOR signaling pathway was also activated, which induces trained immunity. In this in vitro infection model, increased cytokine responses to lipopolysaccharides (LPS) restimulation, higher cell viability, and decreased *Candida albicans* loads were observed.

**Conclusions:**

We have first characterized the transcriptomic profiles of BCG-infected non-adherent THP-1 cells, and first developed a trained immunity in vitro model of the cells.

**Supplementary Information:**

The online version contains supplementary material available at 10.1186/s12866-024-03191-x.

## Background

Tuberculosis (TB), caused primarily by *Mycobacterium tuberculosis* (*Mtb*), remains the world’s leading lethal infectious disease [1]. When aerosols containing *Mtb* are inhaled into the lower lungs, the bacteria first encounter lung-resident alveolar macrophages patrolling the air-lung epithelium interface [2–4]. The phagocytized bacteria are then translocated from the site of infection to nearby lymph nodes, facilitating the presentation of antigens and triggering of adaptive immune responses [2]. However, there is a risk of transmitting the bacteria to other organs via bloodstream infection (BSI), resulting in various forms of extrapulmonary TB (EPTB) [5]. Upon infection, there is a selective recruitment of mycobacteria-permissive monocytes via the CCL2 - CCR2 chemokine axis and the establishment of a growth-permissive niche [6]. Furthermore, mycobacteria can bypass macrophage defenses and escape into monocytes or neutrophils, where they can survive [2, 4, 7]. This is particularly the case when human immunodeficiency virus (HIV) infection impairs the ability of macrophages to control *Mtb* [8].

Monocytes and neutrophils, the major phagocytes in human blood, account for respectively 3–10% and 40–60% of leukocytes [9]. Subsets of monocytes have different functions and their proportions change during *Mtb* infection, disease progression, and treatment [10–13], reflecting the important role of these cells in the host’s fight against *Mtb*. Monocytes (and neutrophils) acquire epigenetic modifications and metabolic reprogramming by in vivo exposure to bacillus Calmette-Guérin (BCG) or *Candida albicans* [14, 15]. The modifications result in the acquisition of innate immune memory, termed trained immunity, which confers nonspecific protection against heterologous pathogens by enhancing secondary immune responses [14–16]. Therefore, an exploration of the interaction between mycobacteria and circulating monocytes directly in vitro could help in gaining a better understanding of this key stage. Heterogeneous primary monocytes or relatively pure sub-populations of interest can be isolated from peripheral blood via sophisticated and time-consuming methods such as magnetic/fluorescence activated cell sorting (M/FACS). Alternatively, the non-adherent monocyte-like cell line THP-1 could be an option.

Due to the non-adherent nature of some monocytes [17], as well as their much lower phagocytic capacities than macrophages [18], thus it is challenging to infect non-adherent monocytes by bacteria (e.g. mycobacteria). It is also difficult to remove free extracellular bacteria from non-adherent (infected) monocytes post infection. Therefore, we aimed to employ non-adherent THP-1 cells and BCG to establish an improved infection protocol and, on the basis of this, investigate the response of THP-1 cells post BCG infection.

## Results

### Infection of non-adherent THP-1

**Fig. 1:**
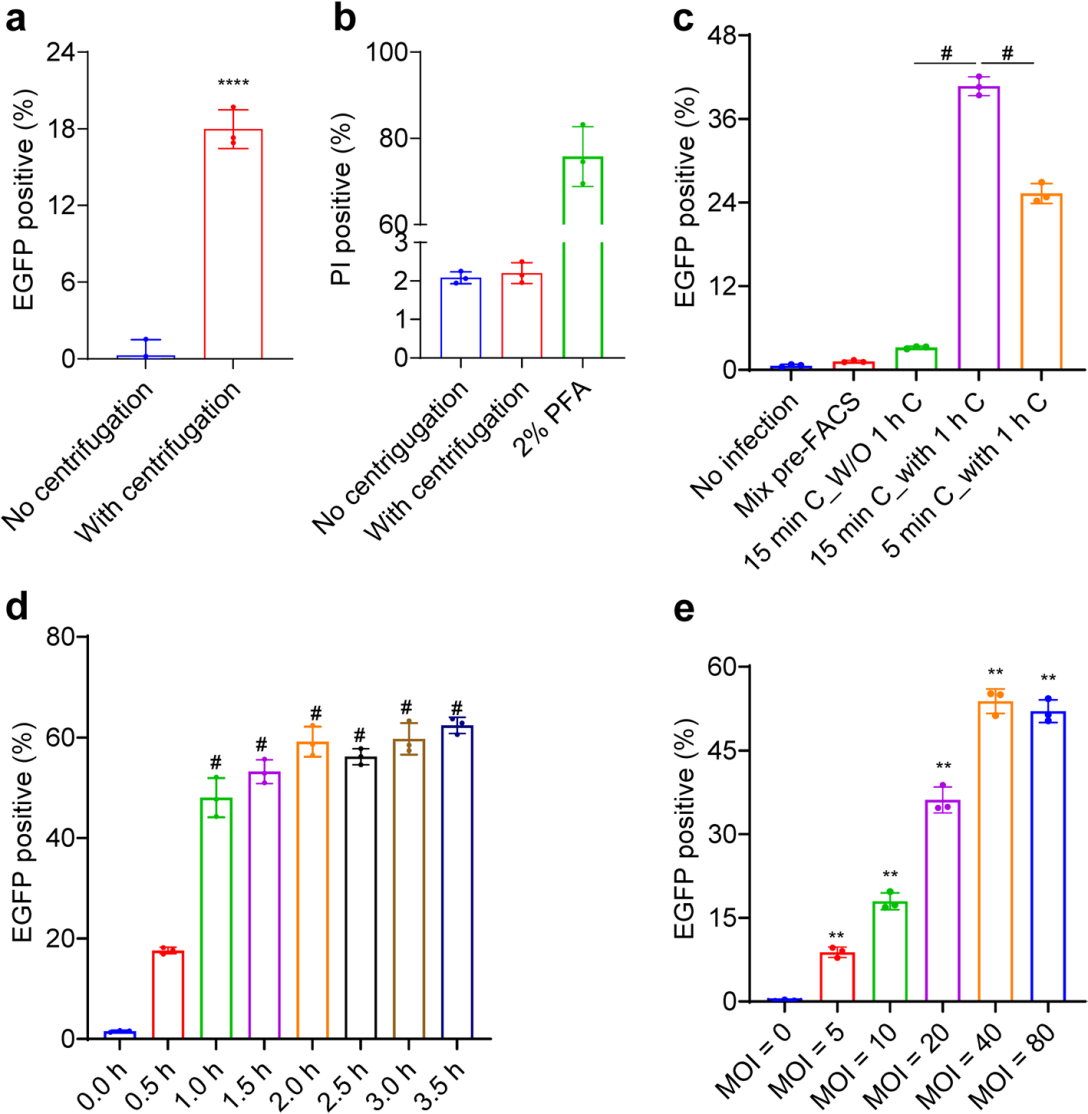
Optimization of parameters for the in vitro infection of non-adherent THP-1 cells by BCG. **a** Centrifugation increases infection efficacy. THP-1 cells (2 × 10^5^ cells/100 µL R10/well) were seeded into 96-well plates, and infected with BCG-EGFP (MOI = 10) with or without centrifugation (200 × *g*, 15 min), and co-cultured for 1 h. EGFP positive cells (FL1-H::FITC) were detected by flow cytometry. **b** No enhanced cell death after centrifugation (200 × *g*, 15 min). Positive control: 2% paraformaldehyde (PFA). **c** The effects of centrifugation and co-culture on infection efficacy. THP-1 cells were seeded into 96-well plates, and infected with BCG-EGFP (MOI = 20) with or without co-culture for 1 h after centrifugation at 200 × *g* for 5 or 15 min. No infection: THP-1 cells without BCG-EGFP infection; Mix pre-FACS: mixing THP-1 cells and BCG-EGFP prior to flow cytometry; 15 min C_W/O 1 h C: centrifugation for 15 min without subsequent 1 h co-culture; 15 min C_with 1 h C: centrifugation for 15 min followed with 1 h co-culture. **d** The effect of co-culture durations on infection efficacy. THP-1 cells were seeded into 96-well plates, and infected with BCG-EGFP (MOI = 20) with different co-culture durations after centrifugation (200 × *g*, 15 min). **e** The effect of MOIs on infection efficacy. THP-1 cells were seeded into 96-well plates and infected with BCG at various MOIs and co-cultured for 1 h after 200 × *g* for 15 min. * *P* < 0.05; ** *P* < 0.01; # *P* < 0.0001. Data shown are the mean ± SD, representative of at least twice repeated experiment

Non-adherent cells and bacteria are in a state of stochastic movement, which is not conducive to the phagocytosis of bacteria by host cells [19, 20]. To establish an efficient infection protocol, constant number of THP-1 cells (i.e. 2 × 10^5^ cells/well of 96-well plate), and then bacteria in differing numbers, were sequentially sedimented onto the bottom of wells in cell culture plates. Centrifugation increased the infection efficacy significantly (Fig. 1a), and no additional cell death was observed (Fig. 1b). The infection efficacy of centrifugation for 15 min at 200 × *g* was significantly higher than centrifugation for 5 mins at 200 × *g* (Fig. 1c). A plateau in infection efficiency (approximately 50% of cells infected) could be reached by co-culture for 1 h after centrifugation (Fig. 1d). The infection efficacy was MOI-dependent, and plateaued approximately at an MOI of 40 (Fig. 1e, Figure S1b and c).

### Removal of non-adherent extracellular bacteria

**Fig. 2:**
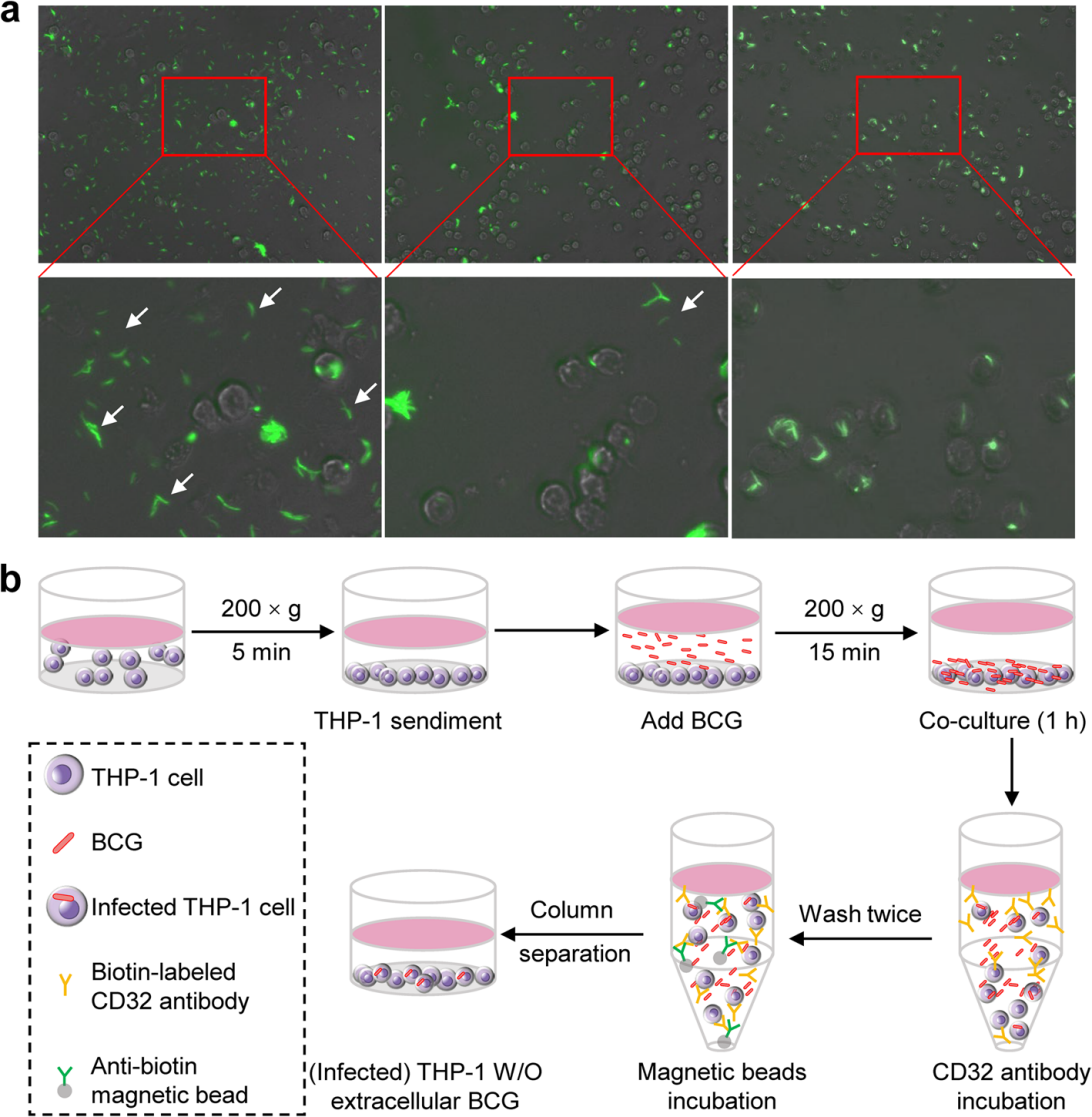
Removal of extracellular BCG by MACS. **a** The removal of extracellular BCG-EGFP (white arrow) by MACS. Left panel: without MACS; middle panel: one round of MACS; right panel: two rounds of MACSs. **b** Schematic of infecting non-adherent THP-1 cells with BCG and MACS for sorting out THP-1 cells from free extracellular BCG

MACS enables cells of interest to be selectively separated. CD32 expresses on the membrane of THP-1 cells [21]. Therefore, we used biotin-labeled anti-human CD32 antibody and anti-biotin magnetic beads to sort out THP-1 cells. The results showed that our MACS strategy could effectively sort out THP-1 cells and remove free extracellular BCG-EGFP (Fig. 2a and Figure S1a). Thus, an efficient infection model/protocol of non-adherent THP-1 cells was established (Fig. 2b).

### The transcriptome of BCG-infected THP-1 cells

RNA-seq was used for transcriptome analysis of BCG-infected non-adherent THP-1 cells. There were significant differences between the BCG group and the BLANK/MOCK groups (Figure S2a). Compared with the BLANK group, the BCG group had 1528 DEGs (|LogFC| ≥ 1, FDR < 0.05), including 1056 up-regulated genes and 472 down-regulated genes (Figure S2b). After BCG infection, the expression of monocyte surface antigens of CD14, CD36, CD40, CD80 and CD86 increased (Table S1), implying cell maturation and activation. Chemokines are critical molecules that recruit immune cells by chemotaxis and activate leukocytes during mycobacterial diseases [22], as seen in the presence of *CCL2–5*, *CCL7–8*, *CCL13*, *CCL20*, *CXCL1–3* and *CXCL8–14* in the up-regulated DEGs. Chemokines bind to their receptors and initiate immune cell migration as well as activation [23].

**Fig. 3:**
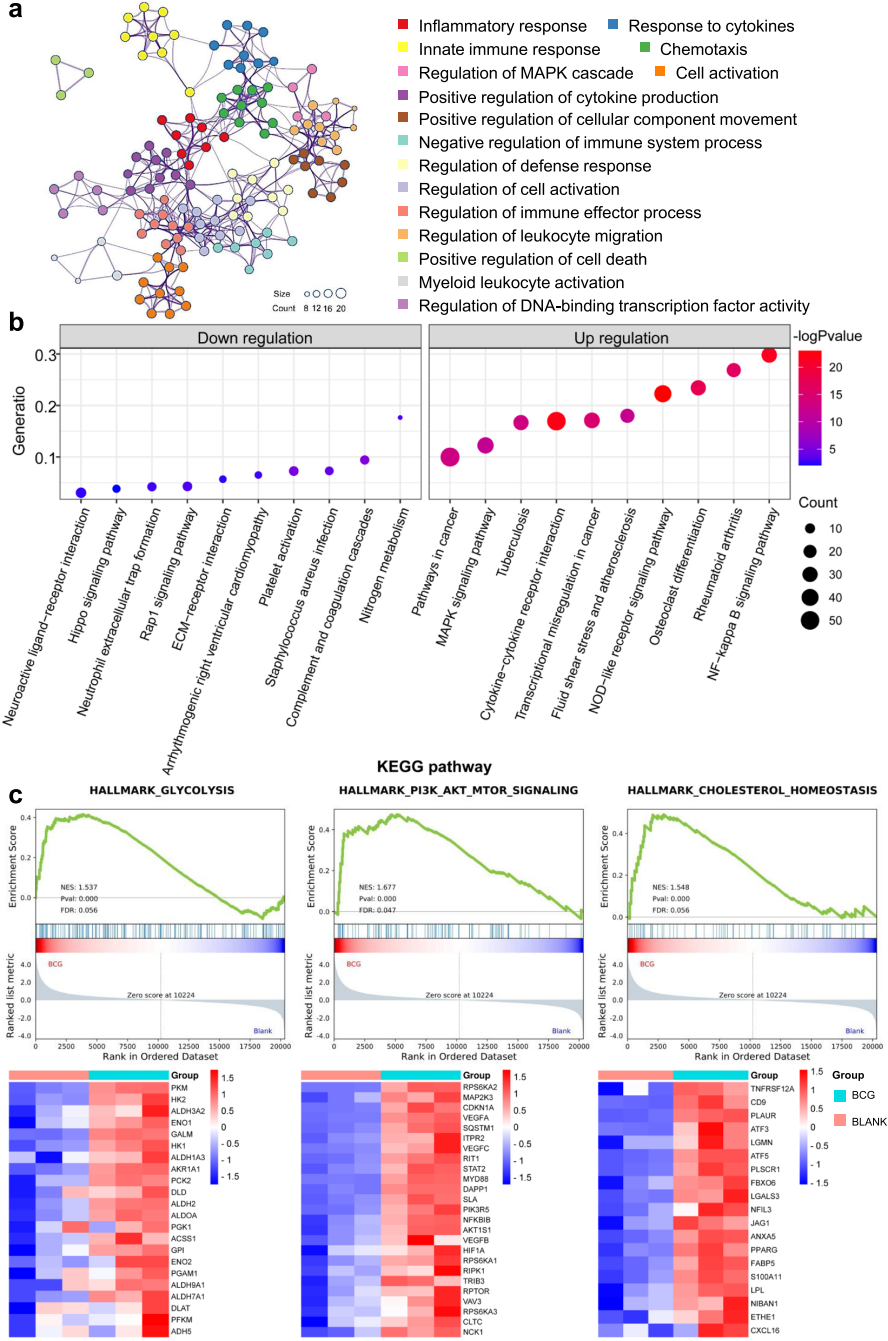
Transcriptomic analysis of BCG-EGFP-infected non-adherent THP-1 cells. **a** The network of GO terms. Top 16 clusters with their representative enriched terms (one per cluster). The “count” is the number of genes present in the given ontology term, colored by cluster ID, where nodes that share the same cluster ID are typically close to each other. **b** The bubble chart of top 10 KEGG pathways. Enriched pathways of up-regulated and down-regulated DEGs were ranked by gene ratio (i.e. mapped gene number/pathway gene number). **c** The GSEA plots associated with trained immunity. Genes are ranked by Signal2Noise in GSEA plot. Gene expression levels decreased from left (red) to right (blue). The matching gene expression heatmaps were shown beneath. Gene expression values were normalized to z-score

Gene ontology (GO) enrichment analyses showed that the down-regulated DEGs were leaded with response to inorganic substance (GO:0010035) and regulation of ion transport (GO:0043269) (Figure S2c); the up-regulated DEGs were leaded with inflammatory response (GO:0006954), positive regulation of cytokine production (GO:0001819) and cellular response to cytokine stimulus (GO:0071345) (Fig. 3a and Figure S2c). Kyoto Encyclopedia of Genes and Genomes (KEGG) pathway enrichment analysis of DEGs showed that the IL-17, NF-kappa B, PI3K-AKt, TNF, Toll-like receptor, NOD-like receptor and MAPK signaling pathways were up-regulated, while Hippo, Rap1 signaling pathways were down-regulated (Fig. 3b and Table S2). GSEA (using hallmark gene sets) analysis of all genes revealed that the inflammatory response (e.g. TNF, IL-6 and IFN-γ, etc.), PI3K-AKT-mTOR pathway, glycolysis, reactive oxygen species pathway, fatty acid metabolism and cholesterol metabolism pathway were activated (Fig. 3c, Figure S2d and Table S3). Glycolysis and cholesterol metabolism have been shown to be involved in trained immunity [24, 25]. The PI3k-Akt-HIF1a-mTOR signaling pathway axis has been shown to be the molecular basis for the induction of trained immunity in monocytes [25]. Therefore, we used this model to study the induction of trained immunity in non-adherent THP-1 cells.

### Short-term BCG-induced trained immunity

**Fig. 4:**
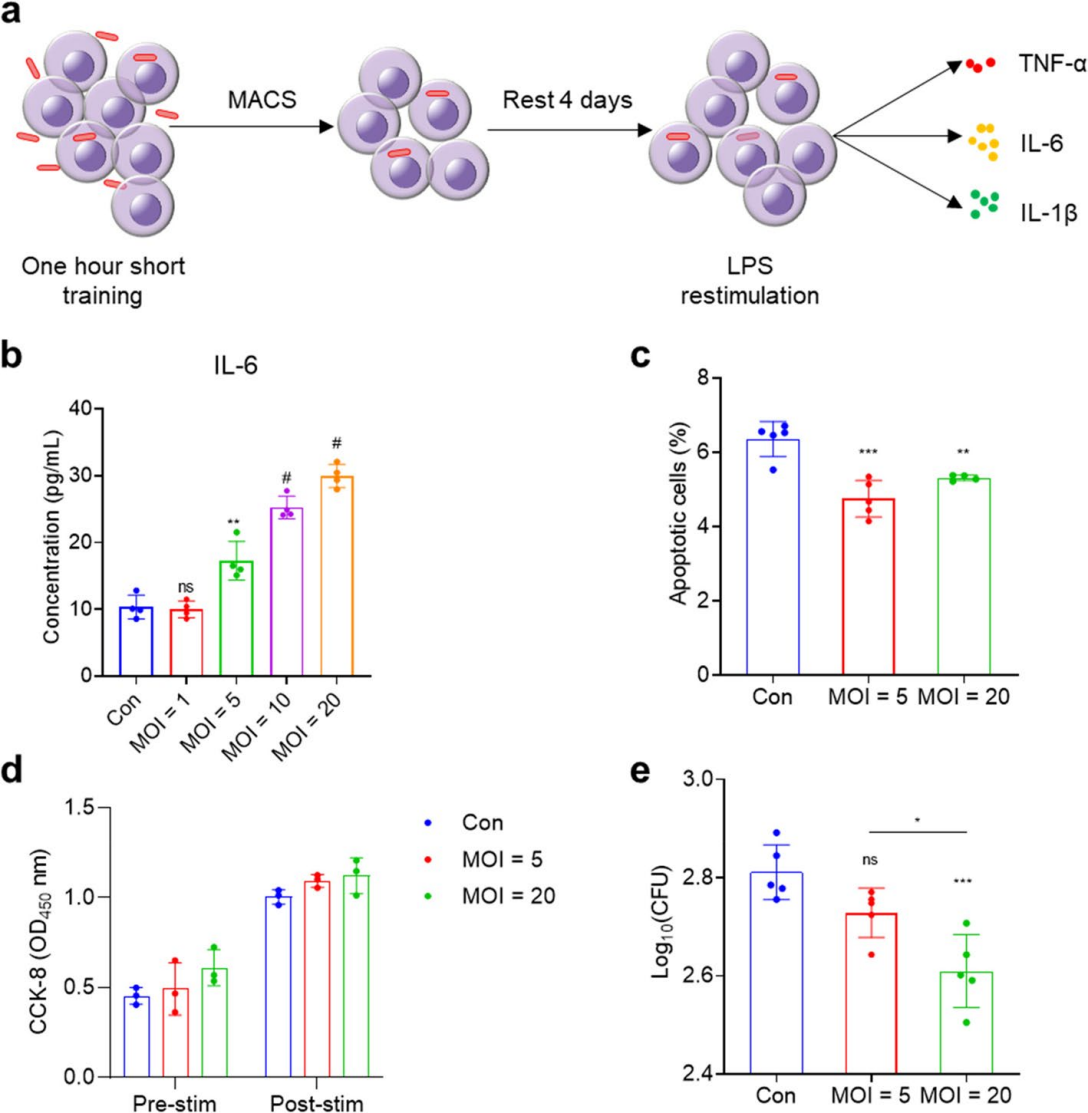
BCG-induced trained immunity in non-adherent THP-1 cells. **a** Schematic of short-term trained immunity. **b** The concentration of IL-6 secreted by trained THP-1 cells after LPS restimulation. **c** The apoptosis measured by flow cytometry 24 h after LPS stimulation. **d** The cell viability of BCG-trained THP-1 cells before and 24 h after LPS stimulation. Pre-stim: before LPS stimulation; post-stim: 24 h post LPS stimulation. **e** The CFUs of *C. albicans* (3 h post infection) after infecting BCG-trained THP-1 cells. ns *P* > 0.05; * *P* < 0.05; ** *P* < 0.01; *** *P* < 0.001; # *P* < 0.0001. Data shown are the mean ± SD, representative of at least twice repeated experiments

The induction of trained immunity by BCG was carried out according to the schematic diagram showed in Fig. 4a. Compared with the control group, BCG training increased IL-6 secretion, and this trend was MOI-dependent (Fig. 4b). The BCG-trained THP-1 cells had increased cell viability than the control cells, even after (lipopolysaccharide) LPS stimulation (Fig. 4d). Consistently, there was decreased apoptosis in THP-1 cells after BCG training (Fig. 4c and Figure S3). Importantly, the trained non-adherent THP-1 cells have lower *C. albicans* loads compared to untrained cells at 3 h post infection (Fig. 4e).

## Discussion

*Mtb* is one of the most common pathogens causing BSI among HIV-infected patients living in high TB-burden settings with a prevalence of nearly 20% and a case-fatality rate approaching 50% [26–29]. Monocytes are the major cell type known to be infected with *Mtb* and play a vital role in immune response against *Mtb* infection [30]. THP-1 cells were widely used to investigate the functions of monocytes and macrophages [31, 32].

The BCG pellets resuspended in R10 were settled to the bottom of the centrifuge tubes after centrifugation at low speeds of 200 × *g* and 500 × *g* for 5 min (Figure S4a). The different buoyant densities of monocytes and mycobacteria have been reported [33, 34]. When using density gradient centrifugation, it was found that both THP-1 cells and BCG located at the same interface between the medium and the Ficoll (Figure S4b). These results indicated that differential density gradient centrifugation was not feasible for removing free extracellular mycobacteria from suspending cells. Finally, we developed a low-speed centrifugation and MACS-based in vitro infection protocol for non-adherent THP-1 cells (Fig. 2b). The model/protocol (Fig. 2b) has the advantages of high infection efficacy, ease of operation, and potentially broad utility in exploring the interaction between other non-adherent cells and pathogenic bacteria.

Although this was the first work to analyze the transcriptome of non-adherent THP-1 cells following BCG infection, the transcriptome of mycobacteria-infected THP-1 cells differentiated (by PMA) macrophages has been previously reported [35–37]. *TNF-α*, *IL-1β*, *CXCL1-2*, and *NFKBIA* were found to be up-regulated after BCG infection [35], and the NOD-like signaling pathway and cytokine-cytokine receptor interaction were enriched in DEGs [36]. The expression levels of genes encoding inflammatory cytokines or cytokine receptors such as *TNF*, *IL-1β*, *IL-32*, *IFNGR* and *TNFR* were also significantly up-regulated in BCG-infected non-adherent THP-1 cells (Table S1). These gene products play important immunomodulatory roles in the early stages of mycobacterial infection [23, 38]. However, the expression level of *IL-10* also increased 5-fold (Table S1). IL-10 can suppress the presentation of antigens, as well as the production of pro-inflammatory cytokines, chemokines, co-stimulatory molecules and adhesion molecules in macrophages and other cell types [39, 40]. Further gene enrichment analysis showed that BCG-infected THP-1 cells were up-regulated in the cytokine-cytokine receptor interaction, NOD-like receptor signaling pathway, chemotaxis, TNF, MAPK, NF-κB, IL-17 and other pathways (Table S2) involved in mycobacteria infection [41, 42]. The NOD-like receptor signaling pathway was also found to be enriched in phorbol 12-myristate 13-acetate (PMA)-differentiated THP-1 macrophages after BCG infection [36].

Intriguingly, the Akt-mTOR-HIF-1a pathway, which could induce aerobic glycolysis as the metabolic basis of trained immunity [43], was also up-regulated in BCG-infected non-adherent THP-1 cells (Fig. 3b). Consistent with our findings (Fig. 4c and d), BCG immunization has been shown to inhibit monocyte apoptosis and increase cell viability [44–46]. The β-glucan-induced trained immunity to protect mice from sepsis has been reported [47], and our results also suggested that the BCG-trained non-adherent THP-1 cells have lower *C. albicans* loads compared to untrained cells (Fig. 4e).

Trained immunity models using primary human blood monocytes have been established, of which the cells are isolated (via CD14-mediated MACS or plastic adhesion), cultured (adherent) and trained in vitro [48, 49], and do not mimic the characteristic suspending/non-adherent state in vivo. It has been shown that shear flow in dynamic culture reduced pro-inflammatory signaling while increasing secretion of the anti-inflammatory cytokine IL-10 and enhancing migration of monocytes [50]. Furthermore, currently used isolation methods affect monocyte function [51–55], and adherent culture also activates monocyte differentiation to macrophages [56]. However, there are no data on BCG-trained immunity using suspending primary monocytes. To the best of our knowledge, all current trained immunity studies involving THP-1 cells have been performed using PMA-differentiated THP-1 macrophages [57–60] rather than using non-adherent THP-1 cells. In this study, we firstly established a trained immunity model of suspending monocytes based on non-adherent THP-1 cells. Although there are studies (albeit limited) reporting differences between non-adherent THP-1 cells and primary monocytes [61, 62], it would be intriguing to study trained immunity and infection of suspending primary monocytes using this model later.

## Conclusions

In summary, an efficient in vitro model/protocol for infecting non-adherent THP-1 cells with BCG by combining low-speed centrifugation, co-culture, and MACS is established, of which the principle is also applicable to other non-adherent cells and bacteria. In addition, we addressed the transcriptomic profiles of non-adherent THP-1 cells infected with BCG, which had not been explored previously to our knowledge. Finally, we used this in vitro model to test the trained immunity of THP-1 cells induced by BCG, which holds in vivo hint.

## Materials and methods

### Bacterial strains and growth conditions

*Mycobacterium bovis* BCG Pasteur was kindly gifted by Xiao-ming Zhang’s lab of Institut Pasteur of Shanghai Chinese Academy of Sciences, China. The cognate BCG-EGFP was constructed via electroporating BCG Pasteur with an EGFP-expressing plasmid (i.e. EGFP was cloned into the plasmid of pMFA41) [63] and being selected on Middlebrook 7H11 agar with kanamycin (final concentration: 50 µg/mL). BCG was grown at 37 °C in liquid Middlebrook 7H9 broth (BD Difco, USA) supplemented with 10% (v/v) oleic acid-albumin-dextrose-catalase enrichment (OADC; BD Difco, USA), 0.5% glycerol and 0.05% Tween-80. The complete medium is referred to as 7H9 hereafter. When necessary, kanamycin was added to a final concentration of 50 µg/mL. *Candida* (*C.*) *albicans* was grown in Sabouraud broth medium (SDB: 1% peptone, 4% dextrose) or on Sabouraud agar medium (SDA: 1% peptone, 4% dextrose, 2% agar) at 37 °C.

### Cell culture

THP-1 cells (ATCC) were grown at 37 °C with 5% CO_2_ in RPMI 1640 (Biological Industries, USA) with 10% fetal bovine serum (FBS; Biological Industries, USA). This complete medium is referred to as R10 hereafter.

### Cell infection

THP-1 cells (2 × 10^5^ cells/100 µL R10/well) were seeded into 96-well plates. Plates were centrifuged (200 × *g*, 5 mins) to sediment cells. Then the BCG-EGFP strain suspended in 100 µL R10 was gently/carefully added into each well without disturbing THP-1 cells. BCG pellets were added at a range of multiplicities of infection (MOI; BCG-EGFP:THP-1 or BCG:THP-1 = 0, 1, 5, 10, 20, 40 or 80). Then the plates were centrifuged once again (200 × *g*, 15 min) to sediment the bacteria. Following that, plates were carefully transferred into a cell culture cabinet and incubated at 37 °C for 1 h or other times required.

### Testing the protocols for removing non-adherent extracellular bacteria

#### Protocol 1

Testing if BCG and THP-1 cells could be physically separated by low-speed centrifugation. BCG cultured in 7H9 was sedimented at 1800 × *g* for 5 min. Then the supernatant was discarded and BCG pellets were gently re-suspended in R10 and sedimented at 500 × *g* or 200 × *g* for 5 min.

#### Protocol 2

 Testing if BCG and THP-1 cells could be physically separated by Ficoll density gradient centrifugation. One mL of Ficoll (TBD, China) was added into a 1.5-mL tube, then gently overlaid with 0.5 mL R10 containing THP-1 cells or BCG. After centrifugation at 400 × *g* for 20 min (acceleration = 5, deceleration = 4). Then the vertical positions of THP-1 cells and BCG layers were visually assessed.

#### Protocol 3

 Testing if BCG and THP-1 cells could be physically separated by MACS. Infected THP-1 cells were suspended in 50 µL R10 containing 1 µL CD32-biotin antibody (Miltenyi Biotec, USA), and incubated at 4℃ for 30 min. Then 150 µL R10 was added and sedimented cells. Cell pellets were washed once with 200 µL R10 and sedimented cells. Cell pellets were re-suspended in 80 µL R10, and the suspension was further added with 20 µL anti-biotin MicroBeads (Miltenyi Biotec). After incubating at 4℃ for 15 min, 2 mL R10 was added and sedimented at 300 × *g* for 10 min. Cell pellets were re-suspended in 500 µL R10 and subjected to MACS according to the user manual. The separated cells were visually observed under fluorescence (confocal) microscopy. R10 was exclusively used across protocol 3 (including staining and MACS) rather than other buffers to avoid undesirable induction of serum-starvation-relevant autophagy [64].

### Analysis of RNA-seq data

Non-adherent THP-1 cells were infected with BCG-EGFP (MOI = 20) as described above, or with a null infection. There were three groups (three repeats/group): group 1/BLANK, null infection and null MACS; group 2/MOCK, null infection and MACS. Group 3/BCG, BCG-EGFP infection and MACS. The three groups were operated identically during the cell/bacteria interaction phase. Subsequently the three groups were cultured further for 24 h. RNA was extracted using RNAiso Plus (TaKaRa, Japan). The subsequent RNA quantification, mRNA capture, and RNA sequencing were performed by GENEWIZ Inc. (Suzhou, China) using a 150 base pairs (bp) paired-end sequencing strategy (roughly 6 Giga bp/sample; Novaseq, Illumina, USA). Image analysis and base calling were conducted using the NovaSeq Control Software (HCS) + OLB + GAPipeline-1.6 (Illumina).

Cutadapt (v1.9.1) was used to remove adapters, bases with phred < 20 and being N, and short reads (i.e. < 75 bp) [65]. Hisat2 (v2.0.1) was used to index reference genome sequences downloaded from ENSEMBL (i.e. GRCh38), and to align the cleaned reads to the indexed genome [66]. DESeq2 Bioconductor package (v1.6.3) was used for differential expression analysis (|LogFC| ≥ 1, FDR < 0.05) [67]. Pathway enrichment analysis was performed using Metascape (https://metascape.org/) [68].

Genes were ranked by Signal2Noise, and used for Gene Set Enrichment Analysis (GSEA) using GSEA v4.2.3 (http://www.broadinstitute.org/gsea) [69]. A hallmark gene set with a positive normalized enrichment score (NES) indicates that the gene set is over-represented at the top of a ranked gene list, and thus indicates a positive correlation (up-regulated expression); whereas a negative NES indicates a negative correlation (down-regulation). |NES| > 1 and *P* value < 0.05 were considered as significant enrichment. The cognate gene expression levels were scaled by Z-score and presented as heatmaps (http://www.bioinformatics.com.cn).

### Short-term trained immunity

THP-1 cells were infected as described above and rested for 4 days. On day 4, THP-1 cells were collected and seeded into 96-well plates (2 × 10^5^ cells/well). The cell viability was detected by the CCK-8 kit (Yeasen, China) before and after stimulation with R10 containing 25 ng/mL LPS (eBioscience, USA) for 24 h. The stimulated cells were collected for an apoptosis assay with the Cell Cycle and Apoptosis Analysis Kit (Yeasen, China), and IL-6 in supernatant was assayed by ELISA (Invitrogen, USA). All tests were performed in accordance with the manufacturers’ instructions. In the nonspecific protection assay, *C. albicans* pellets were collected and re-suspended in PBS, then diluted to 0.8–1 × 10^4^ colony forming units (CFUs)/mL with R10, and 50 µL was added to each well. The plates were centrifuged at 600 × *g* for 5 mins to facilitate the bacteria-cells contact, and incubated at 37 °C for 3 h. Next, cells were sedimented at 600 × *g* for 5 mins, and then 0.1 mL of sterile water was added for 10 min to lyse the cells. A series of dilutions were plated on SDA and colonies were enumerated after incubating at 37 °C for 48 h.

### Statistical analysis

Students’ t test (unpaired, two-tailed) and one-way ANOVA with Tukey’s multiple comparisons were used to assess statistical significance using GraphPad Prism (v8.0.1) (https://www.graphpad.com/).

### Supplementary information

**Additional file 1:** **Table S1.** Inflammation-related genes (BCG *vs* Blank). Table S2. KEGG pathways enrichment analysis of DEGs. **Table S3.** GSEA analysis of hallmark gene sets. **Figure S1.** *In vitro* infection of non-adherent THP-1 cells by BCG-EGFP. (a) The confocal images of THP-1 cells infected with BCG-EGFP. (b) The gating strategy of BCG-infected THP-1 cells. (c) The histogram of THP-1 cells infected with BCG-EGFP with different MOIs. **Figure S2.** Transcriptomic analysis of BCG-infected THP-1 cells. (a) The venn diagram of DEGs among BLANK, MOCK and BCG groups. (b) The volcano plot of DEGs (BCG *vs.* BLANK). (c) The top 20 GO biological processes (BCG *vs.* BLANK). (d) The GSEA plots of inflammation-related pathways. **Figure S3.** BCG-induced trained immunity in non-adherent THP-1 cells. (a) The gating strategy of apoptotic THP-1 cells.  (b) The pseudocolor plot of apoptotic and live THP-1 cells. (c) The histogram of apoptotic THP-1 cells infected with BCG. **Figure S4.** The removal of extracellular BCG. (a) Centrifugation could not separate THP-1 cells and BCG. BCG pellets were re-suspended in R10 and sedimented to the bottom of centrifuge tube at 200 or 500 × g for 5 mins. (b) Density gradient centrifugation using Ficoll could not separate THP-1 cells and BCG. The centrifugation parameter was 400 × g, 20 mins (acceleration = 5, deceleration = 4).

## Data Availability

The RNA-seq data of this study have been deposited in the NCBI Sequence Read Archive with the SRA accession number PRJNA905923.
