# Supplementary material for: Establishment of an in vitro model of monocyte-like THP-1 cells for trained immunity induced by bacillus Calmette-Guérin
Source: BMC Microbiol. 2024 Apr 20;24:130. doi: 10.1186/s12866-024-03191-x (PMC11031977; doi:10.1186/s12866-024-03191-x)
Supplement: Supplementary file 1 — Additional file 1: Table S1. Inflammation-related genes (BCG vs Blank). Table S2. KEGG pathways enrichment analysis of DEGs. Table S3. GSEA analysis of hallmark gene sets. Figure S1. In vitro infection of non-adherent THP-1 cells by BCG-EGFP. (a) The confocal images of THP-1 cells infected with BCG-EGFP. (b) The gating strategy of BCG-infected THP-1 cells. (c) The histogram of THP-1 cells infected with BCG-EGFP with different MOIs. Figure S2. Transcriptomic analysis of BCG-infected THP-1 cells. (a) The venn diagram of DEGs among BLANK, MOCK and BCG groups. (b) The volcano plot of DEGs (BCG vs. BLANK). (c) The top 20 GO biological processes (BCG vs. BLANK). (d) The GSEA plots of inflammation-related pathways. Figure S3. BCG-induced trained immunity in non-adherent THP-1 cells. (a) The gating strategy of apoptotic THP-1 cells. (b) The pseudocolor plot of apoptotic and live THP-1 cells. (c) The histogram of apoptotic THP-1 cells infected with BCG. Figure S4. The removal of extracellular BCG. (a) Centrifugation could not separate THP-1 cells and BCG. BCG pellets were re-suspended in R10 and sedimented to the bottom of centrifuge tube at 200 or 500 × g for 5 mins. (b) Density gradient centrifugation using Ficoll could not separate THP-1 cells and BCG. The centrifugation parameter was 400 × g, 20 mins (acceleration = 5, deceleration = 4). [file 12866_2024_3191_MOESM1_ESM.docx]

**Supplementary Tables and Figures**

**Table S1. Inflammation-related genes (BCG *vs* Blank).**

| **Gene_ID** | **GeneSymbol** | **Log_10_FC** | **Log_10_CPM^a^** | ***P* Value** | **FDR** |
| --- | --- | --- | --- | --- | --- |
| ENSG00000275302 | CCL4 | 8.027468 | 2.592502 | 1.00E-152 | 6.43E-150 |
| ENSG00000169245 | CXCL10 | 7.93818 | 3.375349 | 1.32E-135 | 6.9749E-133 |
| ENSG00000108700 | CCL8 | 7.93258 | -0.02776 | 1.96E-23 | 1.35E-21 |
| ENSG00000156234 | CXCL13 | 5.73024 | -0.28545 | 6.23E-16 | 3.02E-14 |
| ENSG00000138755 | CXCL9 | 5.347318 | -1.97292 | 9.35E-05 | 0.001521 |
| ENSG00000108688 | CCL7 | 5.254941 | -2.02715 | 0.000168 | 0.002595 |
| ENSG00000163734 | CXCL3 | 5.236691 | -0.02708 | 1.74E-23 | 1.20E-21 |
| ENSG00000181374 | CCL13 | 5.182702 | -0.75034 | 1.11E-13 | 4.70E-12 |
| ENSG00000121594 | CD80 | 4.775375 | 0.026828 | 1.05E-22 | 6.98E-21 |
| ENSG00000169248 | CXCL11 | 4.421877 | 0.327376 | 4.49E-27 | 3.46E-25 |
| ENSG00000145824 | CXCL14 | 4.163714 | 0.104772 | 9.41E-17 | 4.78E-15 |
| ENSG00000108691 | CCL2 | 4.115507 | 4.423453 | 7.87E-121 | 3.43E-118 |
| ENSG00000277632 | CCL3 | 4.033628 | 3.472945 | 5.89E-133 | 2.99E-130 |
| ENSG00000169429 | CXCL8 | 3.842865 | 7.658067 | 6.23E-267 | 9.93E-264 |
| ENSG00000163739 | CXCL1 | 3.80978 | 3.053621 | 5.53E-143 | 3.19E-140 |
| ENSG00000115009 | CCL20 | 3.770729 | 0.979739 | 6.63E-36 | 6.65E-34 |
| ENSG00000136634 | IL10 | 5.149698 | -2.08379 | 0.00031 | 0.004502 |
| ENSG00000170458 | CD14 | 3.129018 | 2.827411 | 1.85E-82 | 4.50E-80 |
| ENSG00000081041 | CXCL2 | 3.026582 | 1.78689 | 4.97E-48 | 6.52E-46 |
| ENSG00000271503 | CCL5 | 0.788926 | 6.129517 | 9.92E-46 | 1.25E-43 |
| ENSG00000135218 | CD36 | 2.024784 | 7.287454 | 7.77E-296 | 1.62E-292 |
| ENSG00000101017 | CD40 | 1.916527 | 3.223276 | 1.89E-57 | 3.04E-55 |
| ENSG00000125538 | IL1B | 5.026459 | 4.972702 | 4.88E-163 | 3.48E-160 |
| ENSG00000180871 | CXCR2 | -0.70764 | 4.317446 | 8.62E-19 | 4.77E-17 |
| ENSG00000114013 | CD86 | 1.515824 | 0.908512 | 4.05E-09 | 1.19E-07 |
| ENSG00000008517 | IL32 | 3.170501 | 2.017224 | 5.62E-60 | 9.48E-58 |
| ENSG00000107562 | CXCL12 | 1.480926 | 0.07933 | 3.17E-05 | 0.000564 |
| ENSG00000159128 | IFNGR2 | 1.309779 | 6.337573 | 2.27E-93 | 6.74E-91 |
| ENSG00000232810 | TNF | 1.29363 | 5.40987 | 3.18E-35 | 3.14E-33 |
| ENSG00000027697 | IFNGR1 | 1.17138 | 7.269928 | 4.72E-104 | 1.61E-101 |

^a^CPM: Counts per million

**Table S2. KEGG pathways enrichment analysis of DEGs.**

| **Up or Down** | **Description** | **Log_10_*P*** | **Log_10_(Q-value)** | **InTerm/InList** | **Gene ratio** | **Count** |
| --- | --- | --- | --- | --- | --- | --- |
| Up | NOD-like receptor signaling pathway | -22.996 | -20.457 | 41/184 | 0.222826 | 41 |
| Up | Cytokine-cytokine receptor interaction | -22.2242 | -19.986 | 50/295 | 0.169492 | 50 |
| Up | NF-kappa B signaling pathway | -21.6054 | -19.543 | 31/104 | 0.298077 | 31 |
| Up | Osteoclast differentiation | -17.654 | -15.893 | 30/128 | 0.234375 | 30 |
| Up | Rheumatoid arthritis | -16.3536 | -14.660 | 25/93 | 0.268817 | 25 |
| Up | Transcriptional misregulation in cancer | -14.9869 | -13.562 | 33/193 | 0.170984 | 33 |
| Up | Tuberculosis | -13.3781 | -12.043 | 30/180 | 0.166667 | 30 |
| Up | Pathways in cancer | -13.2302 | -11.922 | 53/531 | 0.099812 | 53 |
| Up | Fluid shear stress and atherosclerosis | -12.026 | -10.809 | 25/139 | 0.179856 | 25 |
| Up | MAPK signaling pathway | -11.7191 | -10.522 | 36/294 | 0.122449 | 36 |
| Up | Amoebiasis | -11.3859 | -10.227 | 21/102 | 0.205882 | 21 |
| Up | Phagosome | -11.1289 | -9.988 | 25/152 | 0.164474 | 25 |
| Up | Cell adhesion molecules | -9.72001 | -8.686 | 23/149 | 0.154362 | 23 |
| Up | Malaria | -9.703 | -8.682 | 14/50 | 0.28 | 14 |
| Up | Human papillomavirus infection | -9.03245 | -8.037 | 34/331 | 0.102719 | 34 |
| Up | Ferroptosis | -8.64927 | -7.678 | 12/41 | 0.292683 | 12 |
| Up | Bladder cancer | -8.64927 | -7.678 | 12/41 | 0.292683 | 12 |
| Up | Staphylococcus aureus infection | -8.28326 | -7.335 | 17/96 | 0.177083 | 17 |
| Up | Human cytomegalovirus infection | -7.48474 | -6.569 | 25/225 | 0.111111 | 25 |
| Up | Apoptosis | -7.42026 | -6.515 | 19/136 | 0.139706 | 19 |
| Down | Complement and coagulation cascades | -5.24063 | -2.702 | 8/85 | 0.094118 | 8 |
| Down | Platelet activation | -4.8932 | -2.655 | 9/124 | 0.072581 | 9 |
| Down | Staphylococcus aureus infection | -3.93898 | -1.877 | 7/96 | 0.072917 | 7 |
| Down | Rap1 signaling pathway | -3.14908 | -1.217 | 9/210 | 0.042857 | 9 |
| Down | Nitrogen metabolism | -3.05669 | -1.217 | 3/17 | 0.176471 | 3 |
| Down | Arrhythmogenic right ventricular cardiomyopathy | -2.73068 | -1.037 | 5/77 | 0.064935 | 5 |
| Down | Neuroactive ligand-receptor interaction | -2.51277 | -0.892 | 11/362 | 0.030387 | 11 |
| Down | ECM-receptor interaction | -2.47727 | -0.892 | 5/88 | 0.056818 | 5 |
| Down | Hippo signaling pathway | -2.03217 | -0.493 | 6/157 | 0.038217 | 6 |
| Down | Complement and coagulation cascades | -5.24063 | -2.702 | 8/85 | 0.094118 | 8 |

**Table S3. GSEA analysis of hallmark gene sets.**

| **Description** | **setSize** | **EnrichmentScore** | **NES** | ***P* value** | **Adjust *P*** | **Q values** |
| --- | --- | --- | --- | --- | --- | --- |
| IL2_STAT5_SIGNALING | 178 | 0.67148 | 2.329413 | 1.00E-16 | 7.14E-16 | 2.26E-16 |
| IL6_JAK_STAT3_SIGNALING | 75 | 0.798373 | 2.54191 | 1.00E-16 | 7.14E-16 | 2.26E-16 |
| INFLAMMATORY_RESPONSE | 168 | 0.787435 | 2.724386 | 1.00E-16 | 7.14E-16 | 2.26E-16 |
| INTERFERON_ALPHA_RESPONSE | 96 | 0.833107 | 2.742838 | 1.00E-16 | 7.14E-16 | 2.26E-16 |
| INTERFERON_GAMMA_RESPONSE | 185 | 0.800902 | 2.790187 | 1.00E-16 | 7.14E-16 | 2.26E-16 |
| KRAS_SIGNALING_UP | 156 | 0.697 | 2.392412 | 1.00E-16 | 7.14E-16 | 2.26E-16 |
| TNFA_SIGNALING_VIA_NFKB | 189 | 0.775544 | 2.704037 | 1.00E-16 | 7.14E-16 | 2.26E-16 |
| COMPLEMENT | 166 | 0.66299 | 2.286977 | 9.30E-16 | 5.81E-15 | 1.84E-15 |
| ALLOGRAFT_REJECTION | 152 | 0.630111 | 2.157635 | 5.37E-12 | 2.69E-11 | 8.49E-12 |
| UV_RESPONSE_UP | 141 | 0.595541 | 2.023069 | 3.68E-09 | 1.67E-08 | 5.28E-09 |
| APOPTOSIS | 147 | 0.594838 | 2.029575 | 7.80E-09 | 3.25E-08 | 1.03E-08 |
| HYPOXIA | 173 | 0.562197 | 1.947863 | 6.19E-08 | 2.38E-07 | 7.51E-08 |
| XENOBIOTIC_METABOLISM | 156 | 0.549814 | 1.887205 | 3.15E-07 | 1.12E-06 | 3.55E-07 |
| P53_PATHWAY | 187 | 0.517652 | 1.802649 | 1.51E-06 | 5.03E-06 | 1.59E-06 |
| COAGULATION | 101 | 0.585341 | 1.938043 | 1.93E-06 | 6.03E-06 | 1.90E-06 |
| TGF_BETA_SIGNALING | 52 | 0.660943 | 2.010861 | 8.14E-06 | 2.40E-05 | 7.56E-06 |
| ANDROGEN_RESPONSE | 90 | 0.56653 | 1.851642 | 3.86E-05 | 0.000107 | 3.38E-05 |
| MTORC1_SIGNALING | 198 | 0.461606 | 1.617034 | 0.000205 | 0.000538 | 0.00017 |
| NOTCH_SIGNALING | 29 | 0.6846 | 1.865211 | 0.00038 | 0.00095 | 0.0003 |
| MYOGENESIS | 142 | 0.46918 | 1.592942 | 0.000764 | 0.001819 | 0.000574 |
| APICAL_JUNCTION | 160 | 0.459776 | 1.581634 | 0.001006 | 0.002286 | 0.000722 |
| REACTIVE_OXYGEN_SPECIES_PATHWAY | 49 | 0.585911 | 1.778544 | 0.001333 | 0.002897 | 0.000915 |
| UV_RESPONSE_DN | 131 | 0.463646 | 1.563195 | 0.001788 | 0.003725 | 0.001176 |
| PI3K_AKT_MTOR_SIGNALING | 95 | 0.474321 | 1.558432 | 0.002461 | 0.004922 | 0.001554 |
| ANGIOGENESIS | 26 | 0.645983 | 1.726544 | 0.004551 | 0.008751 | 0.002764 |
| GLYCOLYSIS | 178 | 0.415562 | 1.441616 | 0.005166 | 0.009566 | 0.003021 |
| HEME_METABOLISM | 170 | 0.41751 | 1.445972 | 0.006166 | 0.011011 | 0.003477 |
| CHOLESTEROL_HOMEOSTASIS | 70 | 0.491402 | 1.542826 | 0.008554 | 0.014749 | 0.004657 |
| FATTY_ACID_METABOLISM | 135 | 0.421938 | 1.4282 | 0.009282 | 0.015469 | 0.004885 |
| UNFOLDED_PROTEIN_RESPONSE | 112 | 0.445965 | 1.4861 | 0.010243 | 0.016521 | 0.005217 |
| APICAL_SURFACE | 33 | 0.568529 | 1.593229 | 0.020269 | 0.03167 | 0.010001 |
| PROTEIN_SECRETION | 94 | 0.423279 | 1.390746 | 0.02707 | 0.041015 | 0.012952 |
| ADIPOGENESIS | 184 | 0.383705 | 1.335239 | 0.029516 | 0.043406 | 0.013707 |
| HEDGEHOG_SIGNALING | 30 | 0.552268 | 1.522685 | 0.039131 | 0.055902 | 0.017653 |
| ESTROGEN_RESPONSE_LATE | 159 | 0.37483 | 1.289383 | 0.058683 | 0.081504 | 0.025738 |
| KRAS_SIGNALING_DN | 99 | 0.394742 | 1.306991 | 0.06402 | 0.086514 | 0.02732 |
| ESTROGEN_RESPONSE_EARLY | 172 | 0.360263 | 1.247576 | 0.07619 | 0.100251 | 0.031658 |
| MYC_TARGETS_V2 | 57 | 0.435889 | 1.342846 | 0.081044 | 0.103903 | 0.032811 |
| SPERMATOGENESIS | 90 | -0.30947 | -1.18946 | 0.113924 | 0.142405 | 0.04497 |
| PANCREAS_BETA_CELLS | 19 | 0.514264 | 1.282603 | 0.156766 | 0.191178 | 0.060372 |
| E2F_TARGETS | 200 | -0.25687 | -1.10055 | 0.167407 | 0.199294 | 0.062935 |

**
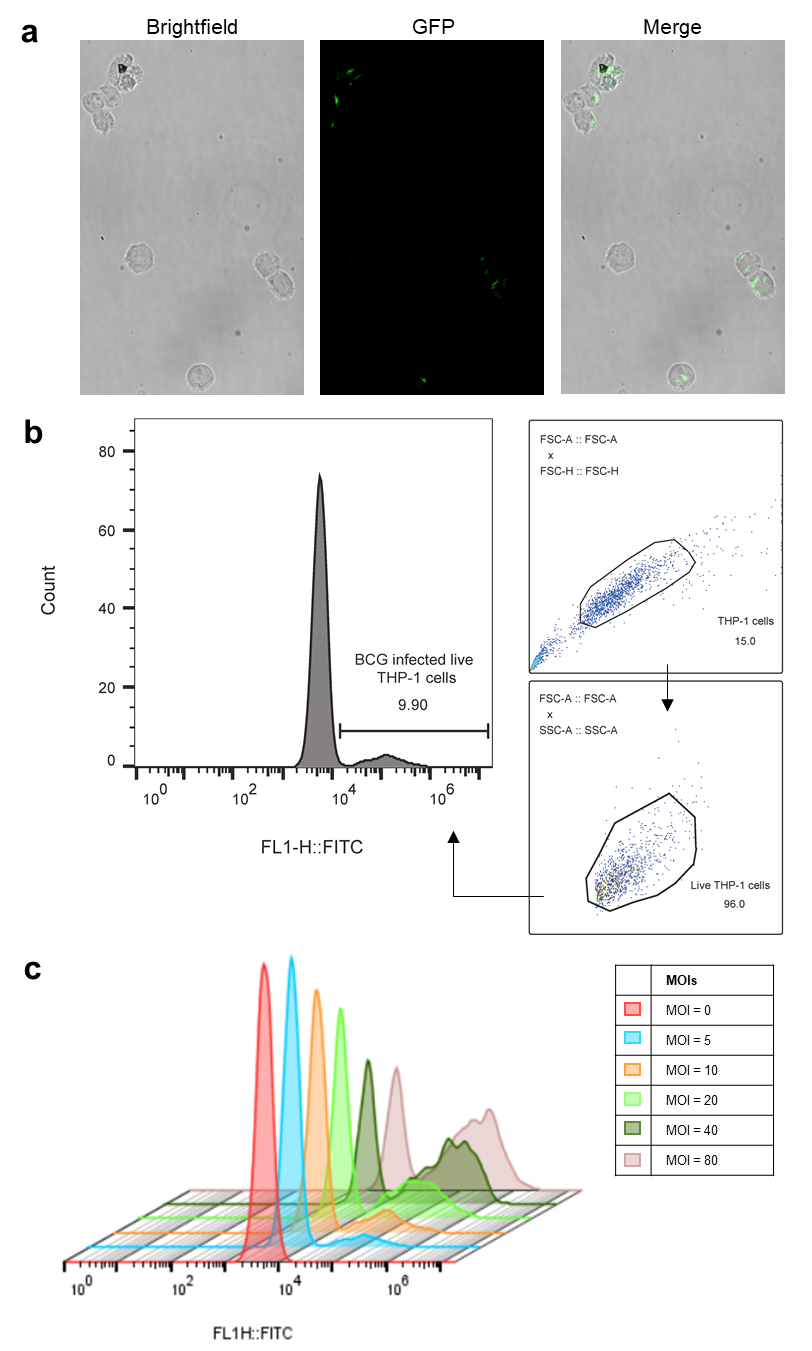
**

**Figure S1. *In vitro* infection of non-adherent THP-1 cells by BCG-EGFP.** (a) The confocal images of THP-1 cells infected with BCG-EGFP. (b) The gating strategy of BCG-infected THP-1 cells. (c) The histogram of THP-1 cells infected with BCG-EGFP with different MOIs.

**
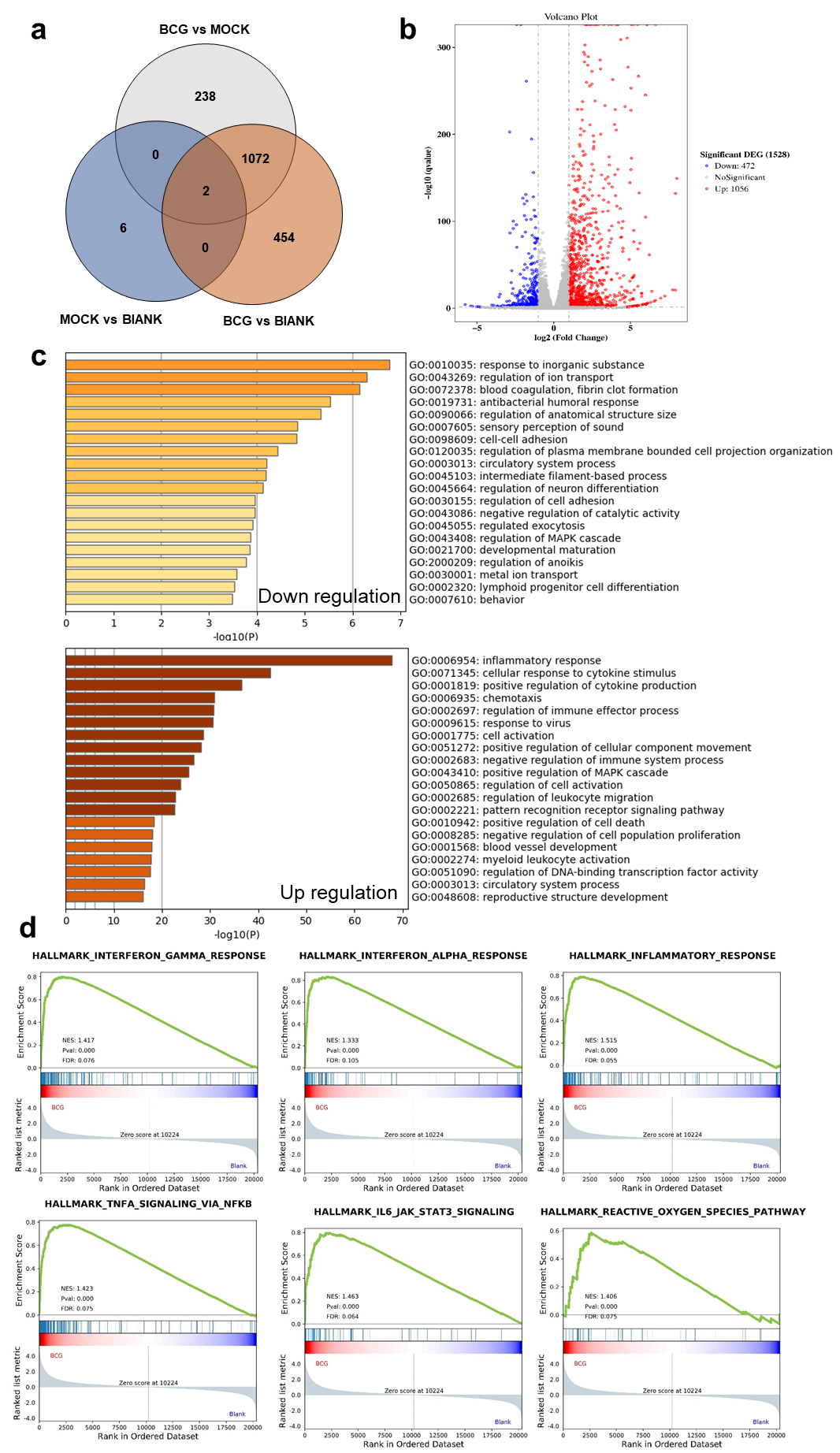
**

**Figure S2. Transcriptomic analysis of BCG-infected THP-1 cells.** (a) The venn diagram of DEGs among BLANK, MOCK and BCG groups. (b) The volcano plot of DEGs (BCG *vs.* BLANK). (c) The top 20 GO biological processes (BCG *vs.* BLANK). (d) The GSEA plots of inflammation-related pathways.


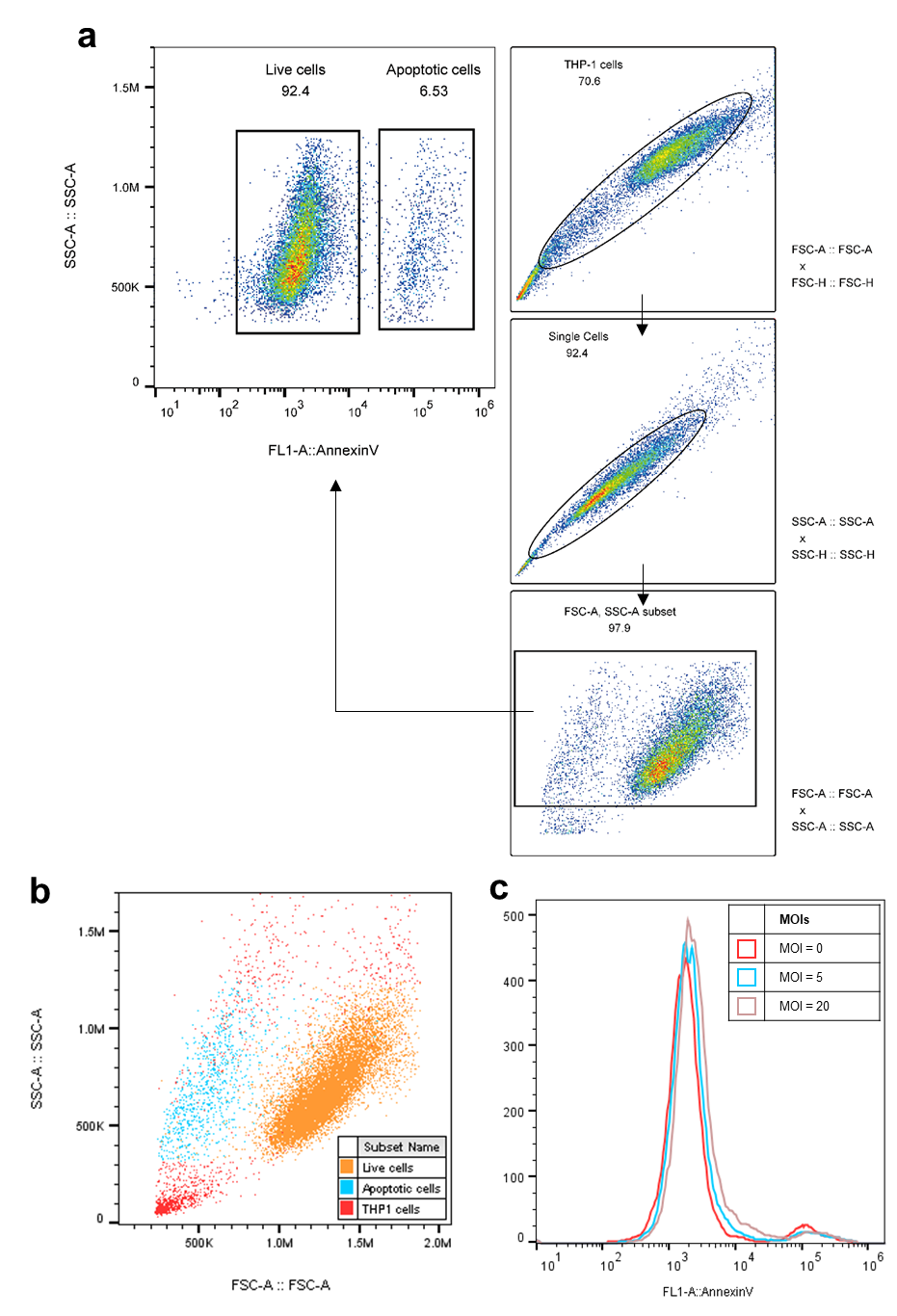


**Figure S3. BCG-induced trained immunity in non-adherent THP-1 cells.** (a) The gating strategy of apoptotic THP-1 cells. (b) The pseudocolor plot of apoptotic and live THP-1 cells. (c) The histogram of apoptotic THP-1 cells infected with BCG.


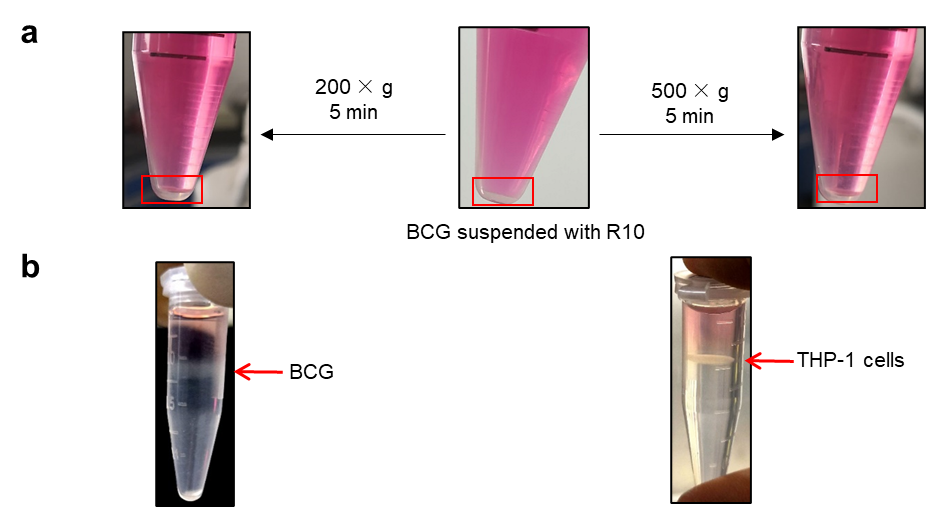


**Figure S4. The removal of extracellular BCG.** (a) Centrifugation could not separate THP-1 cells and BCG. BCG pellets were re-suspended in R10 and sedimented to the bottom of centrifuge tube at 200 or 500 × g for 5 mins. (b) Density gradient centrifugation using Ficoll could not separate THP-1 cells and BCG. The centrifugation parameter was 400 × g, 20 mins (acceleration = 5, deceleration = 4).
